# Effects of Melatonin and Its Underlying Mechanism on Ethanol-Stimulated Senescence and Osteoclastic Differentiation in Human Periodontal Ligament Cells and Cementoblasts

**DOI:** 10.3390/ijms19061742

**Published:** 2018-06-12

**Authors:** Won-Jung Bae, Jae Suh Park, Soo-Kyung Kang, Il-Keun Kwon, Eun-Cheol Kim

**Affiliations:** 1 Department of Oral and Maxillofacial Pathology, School of Dentistry, Kyung Hee University, Seoul 02447, Korea; tarepigu@naver.com; 2 Department of Dentistry, Graduate School, Kyung Hee University, Seoul 02447, Korea; donna10@naver.com; 3 Department of Oral Medicine, School of Dentistry, Kyung Hee University, Seoul 02447, Korea; drcecilia@khu.ac.kr; 4 Department of Dental Materials, School of Dentistry, Kyung Hee University, Seoul 02447, Korea; kwoni@khu.ac.kr

**Keywords:** melatonin, senescence, human periodontal ligament cells, cementoblasts, osteoclast differentiation, PIN1

## Abstract

The present study evaluated the protective effects of melatonin in ethanol (EtOH)-induced senescence and osteoclastic differentiation in human periodontal ligament cells (HPDLCs) and cementoblasts and the underlying mechanism. EtOH increased senescence activity, levels of reactive oxygen species (ROS) and the expression of cell cycle regulators (p53, p21 and p16) and senescence-associated secretory phenotype (*SASP*) genes (interleukin [IL]-1β, IL-6, IL-8 and tumor necrosis factor-α) in HPDLCs and cementoblasts. Melatonin inhibited EtOH-induced senescence and the production of ROS as well as the increased expression of cell cycle regulators and SASP genes. However, it recovered EtOH-suppressed osteoblastic/cementoblastic differentiation, as evidenced by alkaline phosphatase activity, alizarin staining and mRNA expression levels of Runt-related transcription factor 2 (Runx2) and osteoblastic and cementoblastic markers (glucose transporter 1 and cementum-derived protein-32) in HPDLCs and cementoblasts. Moreover, it inhibited EtOH-induced osteoclastic differentiation in mouse bone marrow–derived macrophages (BMMs). Inhibition of protein never in mitosis gene A interacting-1 (PIN1) by juglone or small interfering RNA reversed the effects of melatonin on EtOH-mediated senescence as well as osteoblastic and osteoclastic differentiation. Melatonin blocked EtOH-induced activation of mammalian target of rapamycin (mTOR), AMP-activated protein kinase (AMPK), mitogen-activated protein kinase (MAPK) and Nuclear factor of activated T-cells (NFAT) c-1 pathways, which was reversed by inhibition of PIN1. This is the first study to show the protective effects of melatonin on senescence-like phenotypes and osteoclastic differentiation induced by oxidative stress in HPDLCs and cementoblasts through the PIN1 pathway.

## 1. Introduction

Tooth loss increases with age and this is primarily thought to be due to the breakdown of periodontal tissue around natural teeth as a result of periodontitis [1]. Periodontal disease is characterized by an inflammatory reaction of periodontal tissue that leads to the progressive destruction of tooth-associated structures including alveolar bone, periodontal ligament (PDL) and cementum [2]. PDL cells (PDLCs) located between the tooth cementum and bone play a major role in alveolar bone metabolism in periodontal health and disease because of their ability to secrete factors that regulate the homeostasis of connective and osseous tissue, including inflammatory cytokines and major osteoblast or osteoclast regulators [3,4,5,6]. Cementoblasts are responsible for the production of cementum, which is the gold standard in periodontal regeneration [7]. Therefore, the local microenvironment must be conducive to the recruitment and function of cementoblasts or PDLCs for periodontal regeneration.

Changes in the dynamics of the periodontium occur with age in human tissue and in vitro [8,9]. With increasing age, PDLCs become less proliferative and exhibit decreased mitotic capacity [10], decreased organic matrix production [11] and increased expression of proinflammatory mediators under mechanical stimuli [12,13]. Increased resorption and apposition of cementum occurs with age and may also be responsible for increased irregularity of the cemental surface [14]. PDLCs become senescent following serial subculturing in vitro or following exposure to ionizing radiation and exhibit reduced levels of transcription factors and a decreased ability for osteoblastic differentiation compared to young PDLCs [15,16]. However, the role of cellular senescence in the pathology of age-related periodontal disease is not completely understood.

Melatonin (*N*-acetyl-5-methoxytryptamine) is an endogenous chemical mediator produced primarily by the pineal gland [17]. A free radical scavenger, it exhibits anti-inflammatory and antioxidant activity in vivo and in vitro [18,19,20]. Previously we reported that melatonin had cytoprotective and anti-inflammatory effects on H_2_O_2_-induced cytotoxicity and inflammatory mediators in a model of human chondrocytes and rabbit osteoarthritis via the sirtuin 1 (SIRT1) pathway [21]. Moreover, we demonstrated that it promotes hepatic differentiation of human dental pulp stem cells, which have been clinically implicated in the prevention of liver fibrosis [22].

The decline in melatonin production in aged individuals may be a primary factor contributing to the development of age-related diseases [23]. Melatonin decreases the expression of inflammatory and apoptotic markers [24] and improves age-related insulin resistance [25] and neurodegenerative disease in a senescence-accelerated mouse model [26]. Furthermore, it efficiently inhibits anticancer drug-induced premature senescence in A549 lung cancer cells [27]. It also promotes osteoblastic differentiation via p38 mitogen-activated protein kinase (MAPK) and protein kinase D1 [28] or bone morphogenetic protein/extracellular signal-regulated kinase (ERK)/Wnt signaling pathways [29]. Although its cytoprotective effects have been reported in a model of H_2_O_2_-induced premature senescence in mesenchymal stem cells (MSCs) derived from human bone marrow via the SIRT1-dependent pathway [30], its effects against premature senescence and osteoclastic differentiation induced by oxidative stress in adult differentiated somatic cells such as human PDLCs and cementoblasts remain poorly understood. We investigated the effects and underlying mechanism of action of melatonin in ethanol (EtOH)-stimulated premature senescence and osteoclastic differentiation in human PDLCs and cementoblasts.

## 2. Results

### 2.1. EtOH Treatment Induces Cell Death and Features of Premature Senescence in PDLCs and Cementoblasts

**Figure 1:**
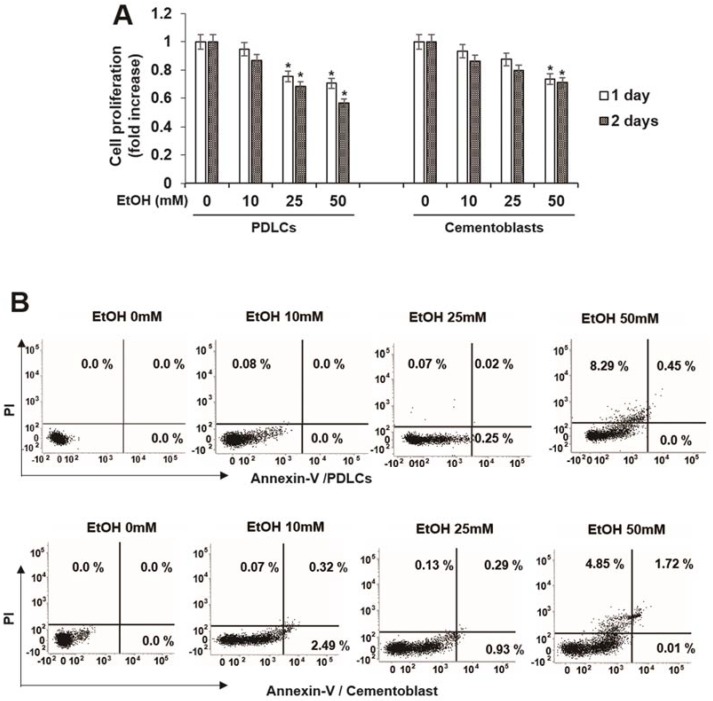
Effect of ethyl alcohol (EtOH) on cell viability (**A**) and cell death in human periodontal ligament cells (HPDLCs) and cementoblast. Cells are incubated with indicated concentration of EtOH for indicated times (**A**) and 3 days (**B**); Cell viability and death were examined by MTT assay and flow cytometry, respectively. These data are representative of three independent experiments. * statistically significant difference compared to the control groups (*p* < 0.05).

To determine the conditions of subcytotoxic stress, we measured cytotoxicity induced by EtOH following 3 days of exposure to doses of EtOH ranging from 10 to 50 mM, based on previous studies performed in osteoblasts [31] and fibroblasts [32]. Cytotoxicity appeared at doses greater than 25 mM EtOH in both PDLCs and cementoblasts (Figure 1A). Therefore, the subcytotoxic doses used in this study were 10–50 mM. To determine whether EtOH inhibits cell survival by inducing apoptosis, we investigated the effects of EtOH on apoptosis. EtOH did not induce early or late apoptosis but induced cell death in PDLCs and cementoblasts (Figure 1B).

**Figure 2:**
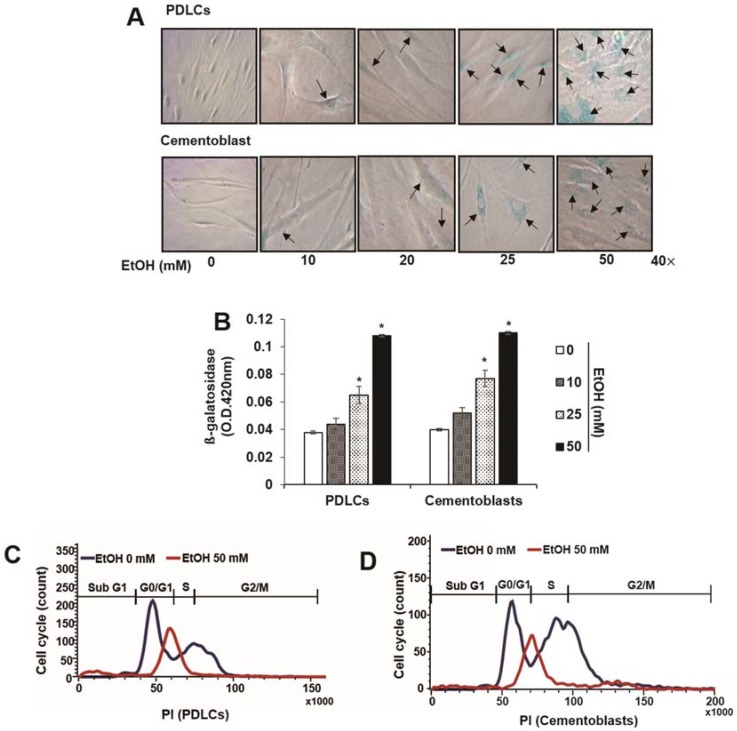
Effect of ethyl alcohol (EtOH) on characterization of cellular senescence by senescence-associated β-galactosidase (β-gal) staining (**A**), β-gal activity (**B**), cell cycle analysis (**C**,**D**) and expression of senescence-associated proteins (**E**) in periodontal ligament cells (PDLCs) and cementoblasts. Cells are incubated with indicated concentration of EtOH for 3 days (**A**–**E**); (**A**,**B**) SA-β-Gal activity was evaluated using a staining kit. Cell cycle and protein analysis were assessed by flow cytometry (**C**,**D**) and Western blot (**E**), respectively. Flow-cytometric frequency histograms of progenitors stained with propidium iodide (PI) for DNA content. These data are representative of three independent experiments. * statistically significant difference compared to the control groups (*p* < 0.05). Arrows in Figure 2A represent β-gal (+) cells.

**Figure 3:**
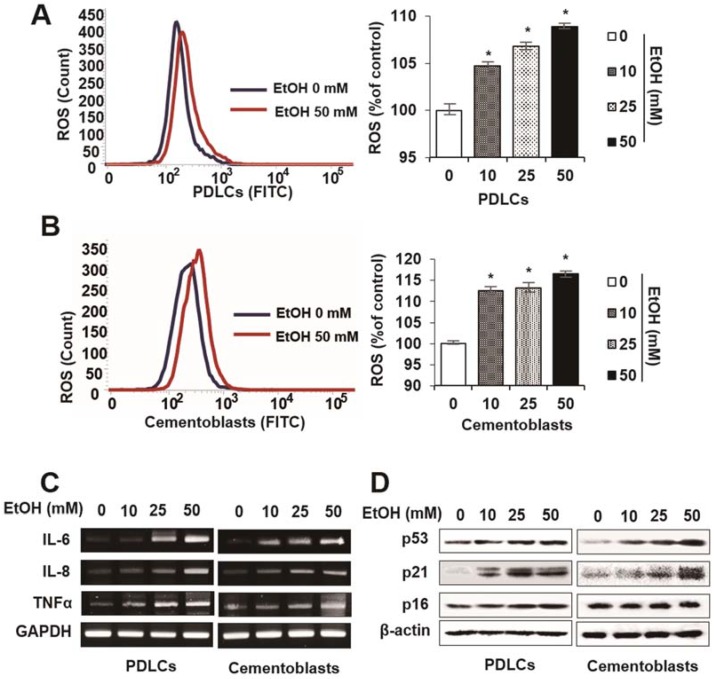
Effect of ethyl alcohol (EtOH) on characterization of cellular senescence by reactive oxygen species (ROS) production (**A**,**B**) and mRNA expression of senescence-associated secretory phenotype (SASP) factors (**C**) in PDLCs and cementoblasts. Cells are incubated with indicated concentration of EtOH for 3 days (**A**–**C**). ROS production and mRNA analysis were assessed by flow cytometry (**A**,**B**) and RT-PCR (**C**), respectively. These data are representative of three independent experiments. * statistically significant difference compared to the control groups (*p* < 0.05).

To determine the effects of EtOH on cellular senescence, we examined senescence-associated β-galactosidase staining and activity in cells treated with EtOH. Following treatment with noncytotoxic concentrations of EtOH, PDLCs and cementoblasts exhibited enlarged nuclei and an expanded cytoplasm, which are morphological changes characteristic of premature senescence (Figure 2A). In addition, β-gal staining and activity increased significantly in a dose-dependent manner following EtOH treatment in both cell types (Figure 2B). To understand cell cycle arrest induced by EtOH treatment, we examined cell cycle profiles by flow cytometry. The proportion of cells treated with EtOH in the G0/G1 phase increased markedly compared to controls in PDLCs and cementoblasts (Figure 2C,D). To verify whether EtOH induces premature senescence in normal cells, we monitored intracellular levels of reactive oxygen species (ROS) and senescence-associated molecular markers. ROS levels increased in a dose-dependent manner in PDLCs and cementoblasts treated with EtOH (Figure 3A,B). Moreover, treatment with EtOH upregulated mRNA expression of major senescence-associated proteins (p53, 16 and p21; Figure 3C) as well as senescence-associated secretory phenotype (SASP) factors (interleukin [IL]-6, IL-8 and tumor necrosis factor [TNF]-α) in PDLCs and cementoblasts (Figure 3D). Together, these results suggest that a sub lethal concentration of 50 mMEtOH causes premature senescence in PDLCs and cementoblasts.

### 2.2. Melatonin Reduces EtOH-Induced Cellular Senescence in PDLCs and Cementoblasts

**Figure 4:**
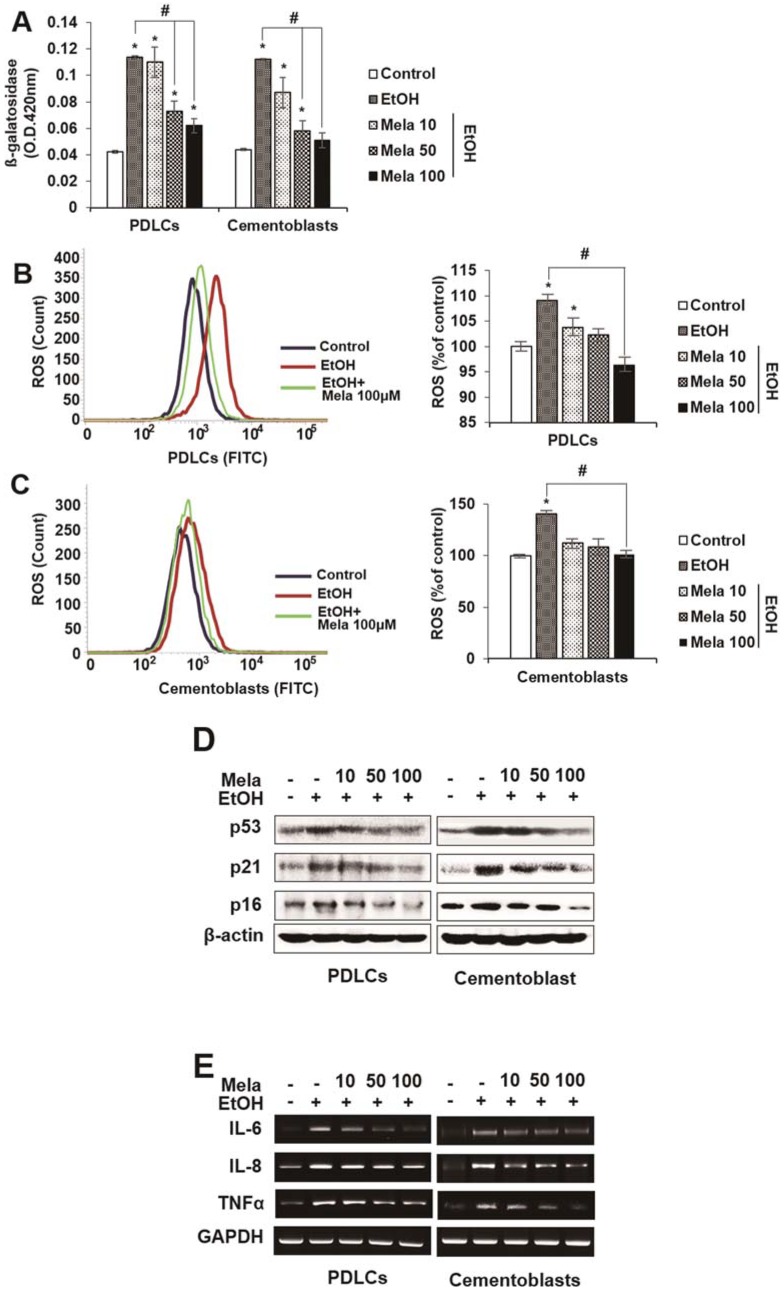
Effect of melatonin on EtOH-induced cellular senescence in PDLCs and cementoblasts. Cells are incubated with indicated concentration of melatonin (μM) and EtOH (25 mM) for 3 days (**A**–**C**). Senescence was examined by β-gal activity (**A**), ROS production (**B**,**C**) and expression of senescence-associated proteins (**D**) and mRNAs (**E**). These data are representative of three independent experiments. * statistically significant difference compared to the control groups (*p* < 0.05). ^#^ statistically significant difference in each group.

Next, we investigated whether melatonin modulates EtOH-induced premature senescence-like phenotypes in PDLCs and cementoblasts. Treatment with melatonin blocked EtOH-induced senescence-like morphological changes, β-gal activity, ROS production and the expression of senescence-associated proteins (p53, 16 and p21) and mRNAs (IL-6, IL-8 and TNF-α) in PDLCs and cementoblasts in a dose-dependent manner (Figure 4A–E).

### 2.3. Involvement of the PIN1 Pathway in the Anti-Senescence Effects of Melatonin

**Figure 5:**
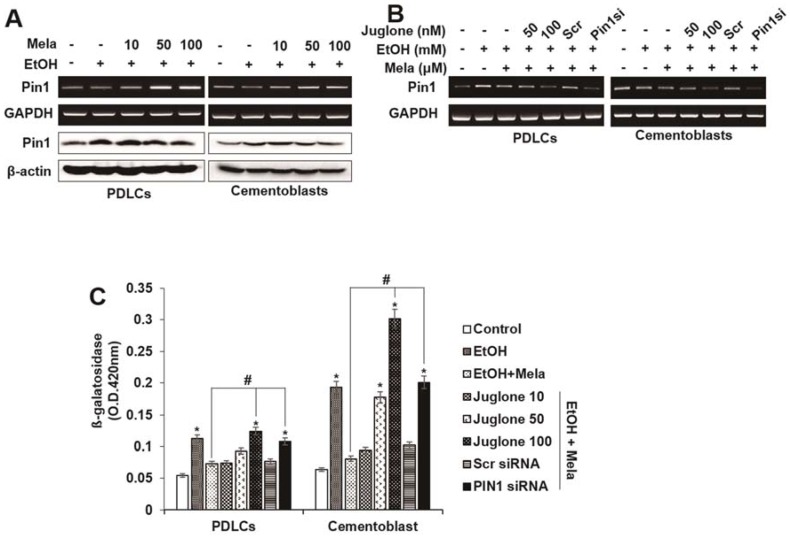

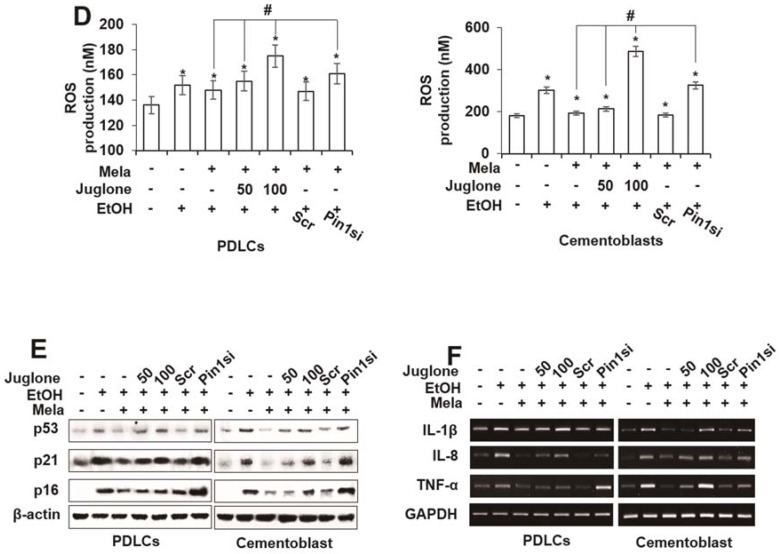
Involvement of PIN1 pathway on effects of melatonin in EtOH-induced cellular senescence of PDLCs and cementoblasts. Cells are pretreated with juglone or PIN1 siRNA and then incubated with melatonin (100 μM) and EtOH (25 mM) for 3 days (**A**–**F**). mRNA and protein expression were accessed by Western blot and RT-PCR (**A**,**B**,**E**,**F**), respectively. Senescence was examined by β-gal activity (**C**), ROS production (**D**,**E**) and expression of senescence-associated proteins (**E**) and mRNAs (**F**). These data are representative of three independent experiments. * statistically significant difference compared to the control groups (*p* < 0.05). ^#^ statistically significant difference in each group.

Because protein never in mitosis gene A interacting-1 (PIN1) may be a molecular target for differentiation and senescence [21,22,33], we investigated whether melatonin affects mRNA or protein expression of PIN1. Melatonin treatment enhanced EtOH-induced PIN1 mRNA and protein expression in PDLCs and cementoblasts in a dose-dependent manner (Figure 5A). To determine whether the anti-senescence effects of melatonin are mediated via a PIN1-dependent pathway, PIN1 expression was knocked down with a small interfering RNA (siRNA) targeting PIN1 (siPIN1) or pretreated with the PIN1 inhibitor juglone. siPIN1 knockdown significantly reversed the inhibitory effects of melatonin on EtOH-induced β-gal activity, ROS production and senescence-associated mRNA or protein expression, whereas the control siRNA showed no effect in PDLCs and cementoblasts. Moreover, pretreatment with juglone showed similar effects as siPIN1 (Figure 5C–F).

### 2.4. Melatonin Reverses EtOH-Suppressed Cementoblastic/Osteoblastic Differentiation

**Figure 6:**
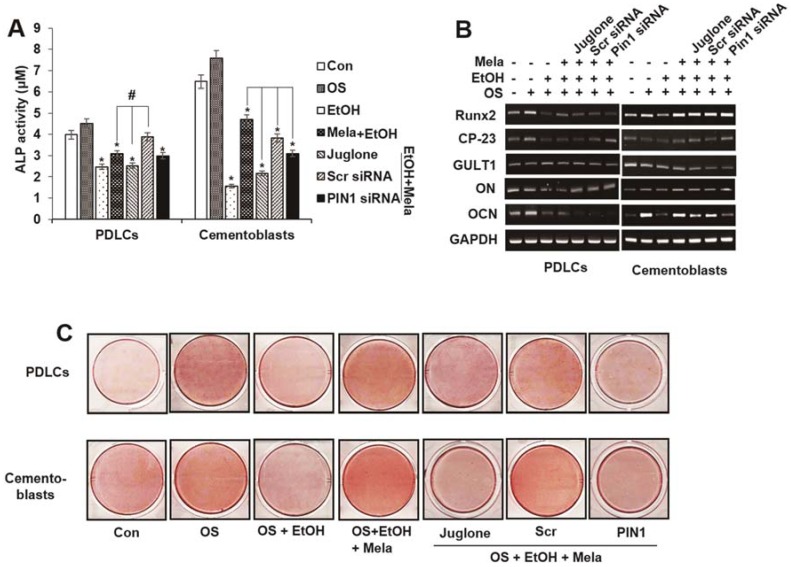
Involvement of PIN1 pathway on effects of melatonin in EtOH-suppressed osteoblastic/cementoblastic differentiation in PDLCs and cementoblasts. Cells are pretreated with juglone (50 nM) or PIN1 siRNA (30 nM) and then incubated with melatonin (100 μM) and EtOH (25 mM) for 14 days (**A**–**C**). Differentiation was accessed by ALP activity (**A**), RT-PCR (**B**) and Alizarin red staining (**C**). These data are representative of three independent experiments. * statistically significant difference compared to the control groups (*p* < 0.05). ^#^ statistically significant difference in each group.

Because the delicate balance between osteoclast-mediated bone destruction and cementoblast-mediated cementum formation is important for maintaining periodontal tissue, we assessed whether EtOH-induced in vitro cementoblastic or osteoclastic differentiation is affected by melatonin. To investigate the effects of melatonin on EtOH-suppressed cementoblastic differentiation, we assessed alkaline phosphatase (ALP) activity, alizarin red staining and cementoblastic marker mRNA expression. As shown in Figure 6, melatonin restored EtOH-suppressed ALP activity, mineralization of nodule formation and mRNA expression of the osteoblastic transcription marker Runx2 and the differentiation markers osteonectin (ON), osteocalcin (OCN) and cementum-derived protein-23 (CP-23) in a dose-dependent manner. However, treatment with the PIN1 inhibitor juglone or siPIN1 reversed the effects of melatonin on EtOH-suppressed cementoblastic differentiation markers, which suggests that PIN1 mediates the rescue effects of melatonin (Figure 6A–C).

### 2.5. Melatonin Reverses EtOH-Induced Osteoclastic Differentiation

**Figure 7:**
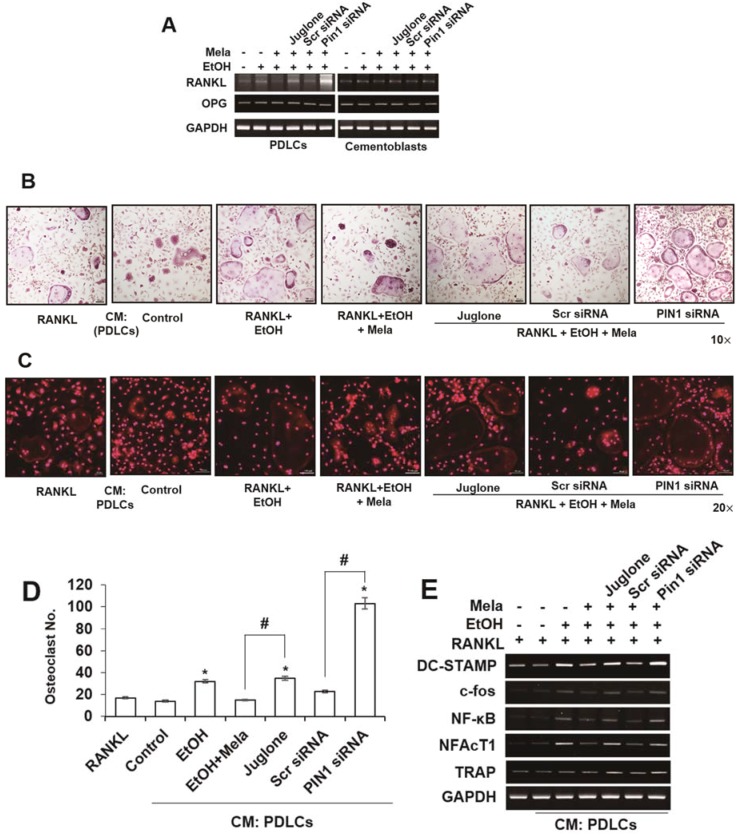

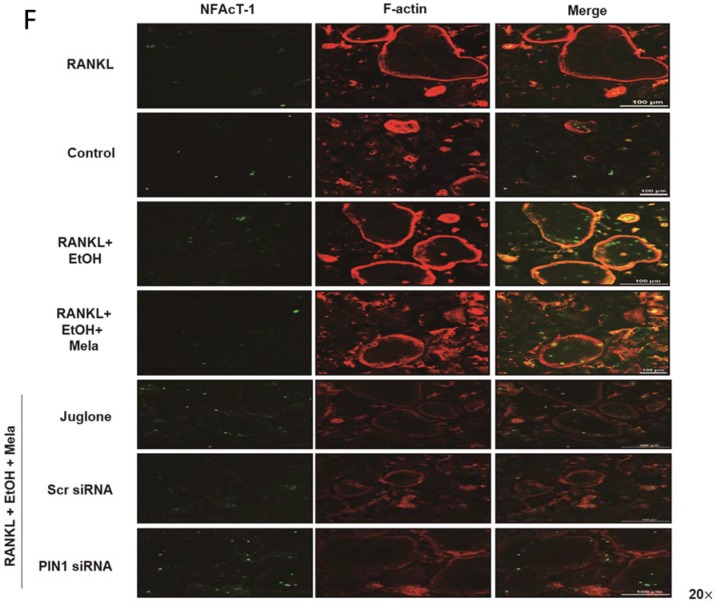
Indirect effects of melatonin on EtOH-induced osteoclastic differentiation in PDLCs and cementoblasts. Cells are pretreated with juglone (50 nM) or PIN1 siRNA (30 nM) and then incubated with melatonin (100 μM) and EtOH (25 mM) for 3 days (**A**) in PDLCs and cementoblasts and conditioned medium (CM) were prepared. The bone-marrow derived macrophage (BMM) cells were incubated with M-CSF (10 ng/mL) and RANKL (50 ng/mL) or 20% CM collected from PDLCs and cementoblasts. After 48 h of culture, the cells were fixed and osteoclast-like cells were identified by TRAP staining; (**B**,**C**) Representative pictures of TRAP staining (**B**) and actin ring (**C**); The numbers of osteoclasts per well were counted (**D**); mRNA expression of osteoclast-specific marker genes was assessed by RT-PCR (**A**,**E**). Representative immunofluorescence of NFATc1 and F-actin expression (**F**) for CM from PDLCs. Similar data were obtained from three independent experiments. Red color is for F-actin. Green color is for NFATc1. Merged color is for F-actin & NFATc1. NFATc1 expression was examined by RT-PCR (**E**) and immunofluorescence (**F**). * statistically significant difference compared to the control groups (*p* < 0.05). ^#^ statistically significant difference in each group.

We investigated the expression of osteoprotegerin (OPG) and receptor activator of nuclear factor (NF)-κB ligand (RANKL) in PDLCs and cementoblasts to determine whether the osteoclastogenic stimulus is affected by melatonin and the inhibition of PIN1. EtOH-induced mRNA expression of RANKL was inhibited by melatonin and this response was reversed following treatment of PDLCs with siPIN1 or juglone. However, OPG mRNA did not change following melatonin and EtOH treatment of PDLCs and cementoblasts (Figure 7A). To examine whether PDLCs and cementoblasts treated with melatonin indirectly regulate osteoclast differentiation, we cultured bone marrow-derived macrophages (BMMs) with CM from PDLCs (Figure 7B–E). Melatonin significantly reduced EtOH-stimulated differentiation of osteoclasts; the number of osteoclasts; actin ring formation; and mRNA expression of osteoclast markers such as dendrocyte-expressed seven transmembrane protein (DC-STAMP), c-fos, NF-κB and nuclear factor of activated T cells (NFAT) c-1. However, treatment with juglone or siPIN1 reversed these effects. Because NFATc1 is a critical transcription factor for the induction of osteoclasts, expression of NFATc1 and F-actin was examined by immunofluorescence. As shown in Figure 7F, NFATc1 and F-actin in the nucleus markedly decreased in cells treated with melatonin compared to cells treated with EtOH. Moreover, PIN1 inhibition reversed the inhibitory effects of melatonin on EtOH-induced NFATc1 translocation (Figure 7F).

**Figure 8:**
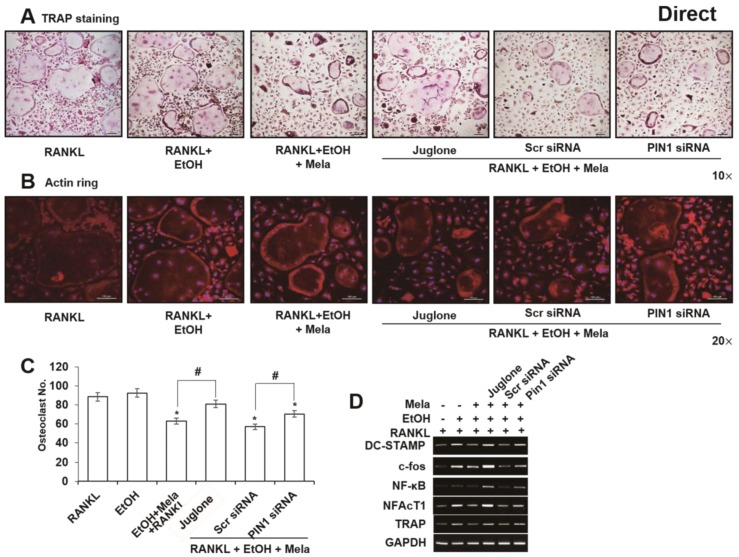
Direct effects of melatonin on EtOH-induced osteoclastic differentiation in BMMs. BMMs were stimulated with RANKL in the presence of juglone (50 nM) or PIN1 siRNA (30 nM), EtOH (25 mM) and melatonin (100 μM) for 5 days. In vitro osteoclatogenesis was accessed by TRAP staining (**A**) and actin ring staining (**B**), counting of osteoclast (**C**), mRNA expression of osteoclast-specific marker genes (**D**). Similar data were obtained from three independent experiments. * statistically significant difference compared to the control groups (*p* < 0.05). ^#^ statistically significant difference in each group.

To determine the direct effects of melatonin on osteoclast differentiation, we examined RANKL-induced osteoclast formation in mouse BMMs. RANKL and EtOH treatment induced the formation of multinucleated osteoclasts but adding melatonin inhibited actin ring formation and the differentiation of BMMs into multinucleated osteoclasts (Figure 8A–C). In addition, melatonin reduced EtOH-stimulated upregulation of tartrate-resistant acid phosphatase (TRAP), DC-STAMP, c-fos, NF-κB and NFATc1 (Figure 8D). Furthermore, treatment with juglone or siPIN1 partly reversed these inhibitory effects on EtOH-stimulated expression of osteoclastic markers (Figure 8E).

### 2.6. AMPK, mTOR and MAPK Signaling Cascades Are Involved in the Effects of Melatonin on EtOH-Mediated Differentiation

**Figure 9:**
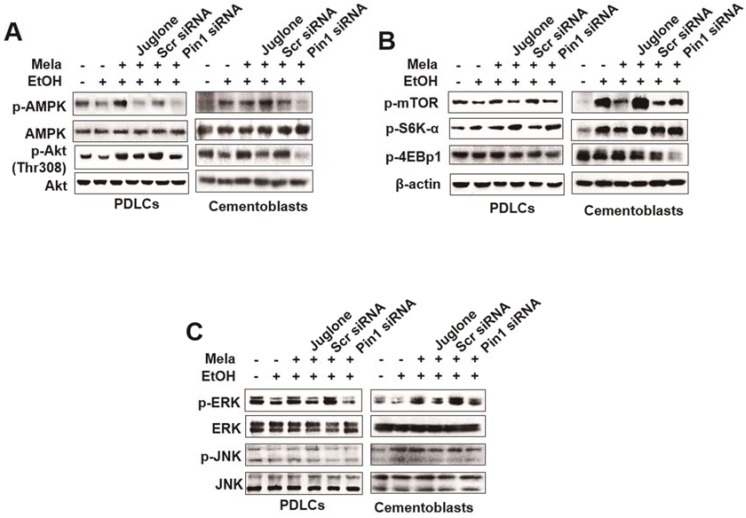
Involvement of AMPK, mTOR and MAPK pathway on effects of melatonin in EtOH-induced senescence or differentiation in PDLCs and cementoblasts. Cells are pretreated with juglone (50 nM) or PIN1siRNA (30 nM) and then incubated with melatonin (100 μM) and EtOH (25 mM) for 60 min (**A**,**B**) and 45 min (**C**). Signal pathways was accessed by Western blot analysis. These data are representative of three independent experiments.

To elucidate the molecular basis of the response to melatonin, we examined its effects on the EtOH-dependent induction of the mammalian target of rapamycin (mTOR) signaling pathway. As shown in Figure 8A, EtOH-dependent inhibition of phosphorylation of AMP-activated protein kinase (AMPK) and Akt (Ser473), which are upstream effectors of mTOR, increased following treatment with melatonin (Figure 9A). However, juglone and siPIN1 treatment inhibited phosphorylation of AMPK and Akt (Figure 9A). Furthermore, melatonin inhibited EtOH-stimulated phosphorylation of mTOR as well as that of ribosomal protein S6K and 4E-bp1, two well-characterized downstream effector molecules of target of rapamycin complex 1 (mTORC1), in PDLCs and cementoblasts (Figure 9B). Moreover, inhibition by juglone or siPIN1 reversed the effects of melatonin on EtOH-induced mTOR activation (Figure 9B). To determine the downstream effectors of melatonin-mediated inhibition of the mTOR pathway, we examined levels of phosphorylated and total MAPK (Figure 9C). Treatment of PDLCs and cementoblasts with melatonin blocked EtOH-induced phosphorylation of ERK but not c-Jun N-terminal kinase (JNK); it did not affect the total protein levels of JNK or ERK. Furthermore, inhibition by juglone or siPIN1 reversed the inhibitory activity of melatonin on EtOH-induced phosphorylation of ERK and JNK (Figure 9C).

## 3. Discussion

Melatonin plays an important role in the bone-healing process because of its antioxidant properties, regulation of bone cells and promotion of angiogenesis [34,35]. However, it is unclear whether it has a positive impact on osteoclastogenesis. In a previous study, exogenous administration of melatonin (5 or 50 mg/kg, daily) for 4 weeks in young mice led to a significant decrease in the number of active osteoclasts on the bone surface without significantly affecting the rate of bone formation [36]. In addition, at 1–500 μM, it causes a dose-dependent reduction in bone resorption activity in vitro, induces the expression of OPG and suppresses the expression of RANKL in mouse osteoblast MC3T3-E1 cells [36]. Furthermore, it has a suppressive effect on osteoclastic and osteoblastic activity in goldfish scales [37]. Melatonin supplementation reduces the number of osteoclasts in an in vivo model of osteoporosis [38] as well as a model of fracture healing [39] but does not significantly affect the number of osteoblasts or osteoclasts against osteoporosis following ovariectomy in rats [40]. Moreover, it does not affect osteoclastogenesis or resorption activity in RAW264.7 cells [41]. Although the beneficial effects of melatonin on periodontal regeneration have been demonstrated in gingival fibroblasts and in an in vivo animal model [42,43], its effects have not been reported in senesced HPDLCs and cementoblasts.

Because the amount of circulating melatonin declines with age, we hypothesized that treatment with melatonin may effectively inhibit cellular senescence and osteoclastogenesis. Because oxidative stress [44,45] and EtOH consumption [31,46] are important causative factors of the induction of periodontitis [44,45] we chose to examine EtOH treatment in HPDLCs and cementoblasts. This is the first study to examine the effects of melatonin and the underlying mechanism of EtOH-induced cellular senescence and osteoclastic differentiation, as well as EtOH-suppressed cementoblastic differentiation, in somatic adult HPDLCs and cementoblasts.

MSCs are a more ethical source of multipotent stem cells capable of differentiating into multiple mesodermal cell lineages such as osteoblasts and myoblasts [47]. Induction of intracellular ROS plays a critical role in the onset of premature senescence of cells upon various genotoxic stresses [48]. Treatment with oxidative agents such as H_2_O_2_ or EtOH at subcytotoxic concentrations for 72 h results in stress-induced premature senescence in normal human somatic cells such as fibroblasts [49,50]. Our results show that subcytotoxic concentrations (10–50 mM) of EtOH induce immature cellular senescence in HPDLCs and cementoblasts, which was confirmed by growth arrest, altered cell morphology, increased β-gal activity and ROS levels and overexpression of senescence-associated genes. These results are consistent with a previous study performed on human fibroblasts [50].

Administration of melatonin reduces H_2_O_2_-induced DNA damage in U-937 cells, which may play an important role in protecting cells from genetic damage due to free radicals [51]. Our results demonstrate that melatonin acts as a potent inhibitor of EtOH-induced cellular senescence, as evidenced by β-gal activity, ROS production and mRNA and protein expression of senescence genes in human PDLCs and cementoblasts. This is consistent with a previous study that showed that melatonin has marked antioxidant and antiaging effects in skeletal muscle in vitro [52].

A number of in vitro and in vivo studies have suggested that melatonin has beneficial effects on bone metabolism, including bone anabolic effects, in which melatonin promotes osteoblastic differentiation [28,29,53]. However, the precise mechanisms and signaling pathways involved in this process, particularly under conditions of EtOH induction, are unknown. To determine its effects on osteoblastic differentiation and mineralization of PDLCs and cementoblasts under EtOH-induced cellular senescence conditions, we evaluated ALP activity as well as the expression of osteogenic or cementogenic genes and mineralization. Melatonin markedly recovered EtOH-suppressed cementoblastic differentiation. Similarly, it recovered H_2_O_2_-suppressed osteoblast differentiation in MC3T3-E1 cells [54].

Phosphorylation of Ser/Thr-Pro motifs can modulate protein function through the induction of conformational changes that are regulated by the unique parvulin-like peptidyl-prolyl cis/trans-isomerase (PIN1), which specifically binds to and isomerizes certain phosphorylated Ser/Thr-Pro motifs in a defined subset of proteins, thereby affecting their function [55]. PIN1 is a ubiquitous enzyme that regulates diverse cellular processes, including growth-signal responses, cell cycle progression and cellular stress and immune responses and aberrant PIN1 function has been implicated in several human diseases [56]. Moreover, PIN1 is present in bone tissue, with the highest levels identified in osteoblasts and osteoclasts [57]. In a previous study, H_2_O_2_-induced oxidative stress increased PIN1 expression in PC12 cells in vitro [27]. In the present study, melatonin potently enhanced EtOH-induced PIN1 mRNA and protein expression in PDLCs and cementoblasts. Moreover, we found that PIN1 reversed the effects of melatonin on EtOH-suppressed osteoblastic or cementoblastic differentiation, which suggests the potential involvement of PIN1 in melatonin-dependent recovery in osteoblastogenesis.

The key cytokines that regulate osteoclastogenesis are RANKL, a stimulator of osteoclast differentiation, activity and survival and OPG, an inhibitor of osteoclastogenesis. In this study, melatonin inhibited EtOH-induced RANKL mRNA expression in PDLCs, which was reversed by inhibition of PIN1. Moreover, it attenuated EtOH-induced osteoclast formation in indirect cultures of PDLC CM as well as direct cultures of BMMs. In addition, treatment with juglone or siPIN1 reversed the effects of melatonin on EtOH-mediated induction of osteoclasts, actin ring formation and expression of osteoclast marker. These results suggest that the direct and indirect anti-osteoclastic differentiation of melatonin involve the PIN1 pathway. The transcription factor NFATc1 has recently been identified as a key regulatory protein that, together with the AP-1 protein c-fos, controls the terminal differentiation of osteoclasts downstream of RANKL signaling [58,59]. Our results demonstrate that melatonin markedly inhibits nuclear translocation of NFATc1 in EtOH-stimulated BMMs and that this response is reversed by inhibition of PIN1. These results suggest that the PIN1 pathway is critical to the anti-osteoclastic effects of melatonin in BMMs.

Oxidative stress, cytokines and growth factors activate the mTOR pathway [60], which is essential for the senescent phenotype [60]. Furthermore, melatonin regulates aging and neurodegeneration via the AMPK/Akt/mTOR pathway [61,62], which has been linked to osteoblastic differentiation in vascular smooth muscle cells [63,64] and MSCs [65]. In the present study, the inhibitory effects of melatonin on EtOH-induced activation of mTOR, AMPK and MAPK signaling were markedly reversed by treatment with juglone or siPIN1. Taken together, our results suggest that melatonin induces cytoprotection and its osteoblastic differentiation effects against EtOH-induced senescence through the downregulation of mTOR, AMPK and MAPK signaling via the inhibition of PIN1. Therefore, melatonin offers a possible approach for treating senescence-associated periodontitis and osteolytic disease.

## 4. Materials and Methods

### 4.1. Cell Culture

Human cementoblasts and periodontal ligament cells (PDLCs) immortalized by transfection with the telomerase catalytic subunit of human telomerase reverse transcriptase (hTERT) were kindly provided by Takashi Takata of Hiroshima University, Japan [66,67]. Cementoblasts and PDLCs were cultured in minimum essential medium alpha (α-MEM) with 10% fetal bovine serum (FBS) plus penicillin G solution (10 U/mL) and streptomycin (10 mg/mL) in a humidified atmosphere of 5% CO_2_ at 37 °C. FBS andα-MEM were purchased from Gibco (Grand Island, NY, USA). To induce differentiation, cells were cultured with osteogenic media (OM, 50 µg/mL ascorbic acid, 10 mM β-glycerophosphate and 10^−7^ M dexamethasone) as described previously [68,69].

### 4.2. Cytotoxicity Assay

Cell viability was evaluated by the 3-(4,5-dimethylthiazolyl-2-yl)-2,5-diphenyltetrazolium bromide (MTT) assay. Briefly, MTT assay solution (1 mg/mL) was added to each 96 well. After a 4 h incubation period (37 °C, 5% CO_2_) the supernatant was removed and the intracellularly stored MTT formazan was solubilized in 200 μL dimethyl sulfoxide for 5 min at room temperature. Optical densities were then measured at 540 nm in a multi-well spectrophotometer.

### 4.3. Senescence-Associated β-Galactosidase (SA-β-gal) Staining

SA-β-gal activity was determined using a SA-β-gal staining kit from Cell Signaling Technology (Beverly, MA, USA) according to the manufacturer’s instructions. Senescent cells were identified as green-stained cells by standard light microscopy. A minimum of 1000 cells was counted in 10 random fields to determine the percentage of SA-β-gal-positive cells.

### 4.4. Reactive Oxygen Species (ROS) Detection

Intracellular ROS generation was measured with the fluorescent probe 5-(and-6)-chloromethyl-2′,7′-dichlorodihydro-fluorescein diacetate, acetyl ester (CM-H2DCFDA; Molecular Probes, Eugene, OR, USA) using a FACScan flow cytometer (Becton Dickinson, San Jose, CA, USA).

### 4.5. Cell Cycle Analysis

Treated cells were harvested and pelleted by centrifugation (400× *g*, 4 °C, 5 min). The cells were fixed with cold 70% ethanol and then stained with PI solution, consisting of 25 mg/mL PI, 10 mg/mL RNase A and 0.1% Triton X-100. After incubation in the dark at 4 °C for 20 min, fluorescence-activated cells were sorted using the FACScan flow cytometer (Becton Dickinson, San Jose, CA, USA) and the data were analyzed using BD CellFIT™ software version 2.0.

### 4.6. FITC-Annexin V/PI Double Staining

After washing twice with PBS, cells (1 × 10^6^) were resuspended in binding buffer (10 mM HEPES/NaOH pH 7.4, 140 mM NaCl and 2.5 mM CaCl_2_) and 1 μg/mL of each Fluorescein isothiocyanate (FITC)-annexin V and PI were added. The mixture was incubated for 10 min in the dark at room temperature and the cellular fluorescence was then measured using flow cytometry.

### 4.7. PIN1 siRNA Transfection

siRNAs were used for gene transient knockdown studies. Cells were incubated in 6-well plates for 24 h and then transfected with control siRNA or PIN1 siRNA (Bioneer, Daejeon, Korea) using Lipofectamine 3000 (Gibco; Invitrogen Ltd., Paisley, UK) according to the manufacturer’s instructions.

### 4.8. ALP Activity and Alizarin Red Staining 

Cells were seeded in 6-well plates at a density of 3 × 10^5^ cells per well and cultured in OM. After 7 days in culture, the cell layer was rinsed with PBS, scraped into 1 mL buffer (10 mM Tris–HCl, 5 mM MgSO_4_, 0.1% Triton X-100, 0.1% NaNO_3_), subjected to three freeze (−20 °C) thaw cycles, sonicated for 5 min to disrupt cell membranes and centrifuged (4000× *g*) at 4 °C for 15 min. ALP activity was measured using *p*-nitrophenyl phosphate (3 mM final concentration) as the substrate for 2 h in 0.7 M 2-amino-methyl-1-propanol, pH 10.3 and 6.7 mM MgCl_2_. Absorbance was measured at 410 nm using an enzyme-linked immunosorbent assay reader (Beckman Coulter, Fullerton, CA, USA). Cells were fixed with 70% ethanol and stained with 1% Alizarin red for 2 h, washed with *deionized water* and observed under a microscope.

### 4.9. Reverse Transcriptase-Polymerase Chain Reaction (RT-PCR)

Total RNA was prepared from cells using TRIzol reagent (Life Technologies, Gaithersburg, MD, USA) according to the manufacturer’s instructions. Reverse transcription of RNA was performed using Accu-Power RT PreMix (Bioneer). cDNA generated by reverse-transcription (2–5 µL) was then amplified using AccuPower PCR PreMix (Bioneer). PCR products were resolved on a 1.5% agarose gel and stained with ethidium bromide.

### 4.10. Western Blot Analysis

Cells were lysed in RIPA buffer (150 mM NaCl, 100 mM Tris–HCl, 1% Tween-20, 1% sodium deoxycholate and 0.1% SDS) with 0.5 mM EDTA, 1 mM PMSF, 10 μg/mL leupeptin, 10 μg/mL aprotinin and 1 μg/mL pepstatin. Proteins were resolved in sodium dodecyl sulfate-polyacrylamide gel electrophoresis (SDS-PAGE) and transferred to nitrocellulose membranes and probed with specific antibodies. Antibodies specific for and p53, p21, p16, phosphor-AMPK, AMPK and actin were obtained from Santa Cruz Biotechnology (Santa Cruz, CA, USA). Antibodies against phospho-mTOR, phospho-S6K, phosphor-4Ebp1, phosphor-Akt, Akt, phospho-ERK (p-ERK), ERK, phospho-p38, p38, phospho-JNK (p-JNK) and JNK were purchased from Cell Signaling Technology (Danvers, MA, USA). The immunoreactive protein complexes were detected by enhanced chemiluminescence (Amersham Bioscience, Boston, MA, USA).

### 4.11. Preparation of Conditioned Medium

PDLCs and cementoblasts (2 × 10^6^) were seeded on 100-mm culture dishes and cultured for 9 days (pre-treated EtOH for 3 days and then change the media with regents for 6 days). To obtain conditioned medium (CM) from these cells, cultured PDLCs and cementoblasts were incubated with fresh α-MEM (without FBS and regents) for the last 24 h. To normalize the differences between cell densities due to proliferation during the culture period, cells from each plate and determined total DNA content/plate (spectrophotometric absorbance, 260 nm) were collected. The CM was then normalized for DNA content between samples by the addition of α-MEM medium.

### 4.12. In Vitro Osteoclast Differentiation

Mouse bone marrow-derived macrophage (BMM) obtained from 8-week-old female ICR mice (Charles River Laboratories, Seoul, Korea) were used as osteoclast precursor cells. BMM cells were seeded in culture plates and cultured in the presence of 30 ng/mL macrophage colony stimulating factor (M-CSF) for 2 days. Induction of differentiation to osteoclasts was achieved by culturing the cells either with 100 ng/mL RANKL (Peprotech, Rocky Hill, NJ, USA) and 30 ng/mL M-CSF (Peprotech) for 2~4 days. Osteoclasts were identified by staining for tartrate-resistant acid phosphatase (TRAP) activity using an acid-phosphatase kit (Sigma-Aldrich, St. Louis, MO, USA) as per the manufacturer’s instruction. The percentage of TRAP positive multinuclear cells among the total cells and the number of mononuclear cells containing more than three nuclei were scored.

### 4.13. Immunocytochemistry

For immunofluorescence analysis, treated cells were fixed and permeabilized with 4% paraformaldehyde for 20 min followed by 0.1% Triton X-100 for 15 min, respectively. After washing in PBS buffer, slides were blocked with 1% normal goat serum for 1 h and then incubated with a mouse monoclonal anti-human NFAcT1 antibody (Biolegend, CA, USA) for overnight at a 1:200 dilution. After rinsing with PBS, the cells were incubated with a fluorescein isothiocyanate (FITC) conjugated goat anti-mouse immunoglobulin (Invitrogen, Carlsbad, CA, USA) for 1 h at a 1:400 dilution and then counterstained with 10 μg/mL 4′,6-diamidino-2-phenylindole (DAPI) and rhodaminephallodidin (Invitrogen) at 1:400 dilution for detection of nuclei and cytosol. Fluorescent images were obtained by laser scanning confocal microscopy (Leica, Wetzlar, Germany).

### 4.14. Statistical Analysis

Statistical analyses of data were performed by one-way analysis of variance (ANOVA) using SPSS software (v20.0; SPSS, Chicago, IL, USA). Differences between groups were determined by unpaired Student’s *t*-test. Values of *p*< 0.05 were accepted as an indication of statistical significance.
